# RNA-CODE: A Noncoding RNA Classification Tool for Short Reads in NGS Data Lacking Reference Genomes

**DOI:** 10.1371/journal.pone.0077596

**Published:** 2013-10-25

**Authors:** Cheng Yuan, Yanni Sun

**Affiliations:** Department of Computer Science and Engineering, Michigan State University, East Lansing, Michigan, United States of America; The Scripps Research Institute, United States of America

## Abstract

The number of transcriptomic sequencing projects of various non-model organisms is still accumulating rapidly. As non-coding RNAs (ncRNAs) are highly abundant in living organism and play important roles in many biological processes, identifying fragmentary members of ncRNAs in small RNA-seq data is an important step in post-NGS analysis. However, the state-of-the-art ncRNA search tools are not optimized for next-generation sequencing (NGS) data, especially for very short reads. In this work, we propose and implement a comprehensive ncRNA classification tool (RNA-CODE) for very short reads. RNA-CODE is specifically designed for ncRNA identification in NGS data that lack quality reference genomes. Given a set of short reads, our tool classifies the reads into different types of ncRNA families. The classification results can be used to quantify the expression levels of different types of ncRNAs in RNA-seq data and ncRNA composition profiles in metagenomic data, respectively. The experimental results of applying RNA-CODE to RNA-seq of Arabidopsis and a metagenomic data set sampled from human guts demonstrate that RNA-CODE competes favorably in both sensitivity and specificity with other tools. The source codes of RNA-CODE can be downloaded at http://www.cse.msu.edu/~chengy/RNA_CODE.

## Introduction

Noncoding RNAs (ncRNAs), which function directly as RNAs without translating into proteins, play diverse and crucial roles in many biochemical processes. For example, tRNAs and rRNAs aid protein synthesis. SnoRNAs guide rRNA modifications. MicroRNAs (miRNAs) regulate gene expression [1]. Short interfering RNAs (siRNAs) involve in gene silencing in RNAi process [2].

In particular, the development of next-generation sequencing (NGS) technologies sheds light on more sensitive and comprehensive ncRNA annotation. Deep sequencing of transcriptomes of various organisms has revealed that a large portion of transcriptomic data cannot be mapped back to annotated protein-coding genes in the reference genome, indicating that those transcripts may contain transcribed ncRNAs [3]. Total RNA-seq and small RNA-seq data generated by numerous transcriptomic sequencing projects are still accumulating rapidly. Identifying different types of ncRNAs and quantifying their expression levels in different tissues, conditions, and developmental stages have generated new knowledge about functions of ncRNAs. Besides RNA-seq data, ncRNA identification is also important for analyzing metagenomic data, which contain sequenced metagenomes from various environmental samples. For example, 16s rRNA classification [4], [5] and assembly [6], [7] is a fundamental step for studying phylogenies in a sample. NCRNA annotation is, therefore, an important component in post-NGS analysis.

There are two different ncRNA identification problems for NGS data. One focuses on identifying homologs of annotated ncRNAs, such as tRNA, rRNAs, snoRNAs, and many types of miRNAs. Some example applications include comparing expression level changes of let-7 miRNA genes in different developmental stages of *C*. elegans [8], identifying all homologs to annotated miRNAs in the small RNA-seq data of a non-model species [9], and 16s rRNA annotation in metagenomic data [6]. These studies aim to annotate all known ncRNAs or novel members of characterized ncRNA families. The second category focuses on reporting novel ncRNA genes. One possible strategy is to cluster sequences and then apply *de novo* ncRNA gene finding tools such as RNAz [10]. This work belongs to the first category.

The state-of-the-art method for ncRNA homology search is still based on comparative ncRNA identification, which searches for ncRNAs through evidence of evolutionary conservation. As the function of an ncRNA is determined not only by its sequence but also by its secondary structure, which contains interacting base pairs, such as Watson-Crick base pairs and G-U base pairs, successful ncRNA search should take advantage of both sequence and secondary structural similarity. A number of such tools are available such as a general ncRNA search tool Infernal [11] and specialized tools for tRNA [12] and snoRNA [13] etc. However, most existing homology search strategies use complete secondary structure of annotated ncRNAs and are not optimized for NGS data. When applied to short and fragmentary sequences, these tools generate marginal scores and thus cannot distinguish reads sequenced from ncRNAs or other regions. To our best knowledge, trCYK [14] is the only tool that conducts homology search for fragmentary reads sequenced from various types of ncRNAs. However, using it alone tends to incur high false positive rate according to our experimental results.

It is worth noting that although NGS platforms are producing longer reads, many reads sequenced from ncRNAs are still fragmentary. First, many ncRNAs are very long, including mRNA-like long ncRNAs [15], 16s rRNAs [5], etc. Second, the biogenesis shows that some types of small ncRNAs are cleavaged into short products (such as mature miRNAs from their precursors). The sizes of these short products are not increasing with read length.

In order to apply existing ncRNA identification tools to NGS data, read mapping or *de novo* sequence assembly tools are usually applied first to connect short reads into contigs. When the reference genome is available, short reads can be mapped back to the reference genome. Existing ncRNA identification tools can then be applied to the blocks containing overlapping reads along the reference genome. When there is no quality reference genome available, which is often the case for metagenomic data and RNA-seq data of non-model organisms, *de novo* sequence assembly tools can be employed first to connect fragmentary reads into contigs. However, using sequence assembly tools as the first step is not always ideal for ncRNA classification.

First, the quality of read assembly deteriorates significantly in complicated NGS data sets [16]. Different sequence assembly tools generate different sets of contigs. There is no consensus on the best assembly tool. Error-containing contigs often affect down-stream analysis.

Second, successful *de novo* assembly requires relatively high sequencing depth, which is difficult to achieve for many ncRNAs. It is shown [17], [18] that the transcription levels of many types of ncRNA genes are low and condition-dependent. Often it is difficult to foresee which ncRNA genes are lowly transcribed. Thus there lacks information to optimize the parameters of *de novo* assembly tools to produce complete or partial ncRNAs of highly divergent expression levels or abundance.

Third, some types of ncRNAs are cleaved during the maturation process (mature miRNAs). The observed reads sequenced from these ncRNAs do not share any overlap and cannot be assembled. Thus, there is a need for an alternative and better ncRNA search tool for NGS data lacking reference genomes.

In this work, we introduce a comprehensive ncRNA classification tool for short reads: *RNA-CODE*, which is specifically designed for ncRNA identification in NGS data sets that lack reference genomes. Given a set of short reads, RNA-CODE classifies the reads into different types of ncRNA families. The classification results can be used to quantify the expression levels of different types of ncRNAs in RNA-seq data and ncRNA composition profiles in metagenomic data, respectively. RNA-CODE integrates secondary structure based homology search with family-specific *de novo* assembly. The parameters of *de novo* assembly tools can be adjusted in a family-specific fashion.

The remaining of this manuscript is organized as follows. The Methods section describes the design rationale of RNA-CODE and the three main stages. The Results section benchmarks RNA-CODE with other ncRNA classification frameworks. We present experiments results on real metagenomic data and RNA-seq data. For the small-scale metagenomic data, we compare RNA-CODE with Metaxa [4] on 16s rRNA read classification. Then, we compare RNA-CODE with *de novo* sequence assembly on small RNA-seq data annotation of a well-annotated organism.

## Methods

**Figure 1:**
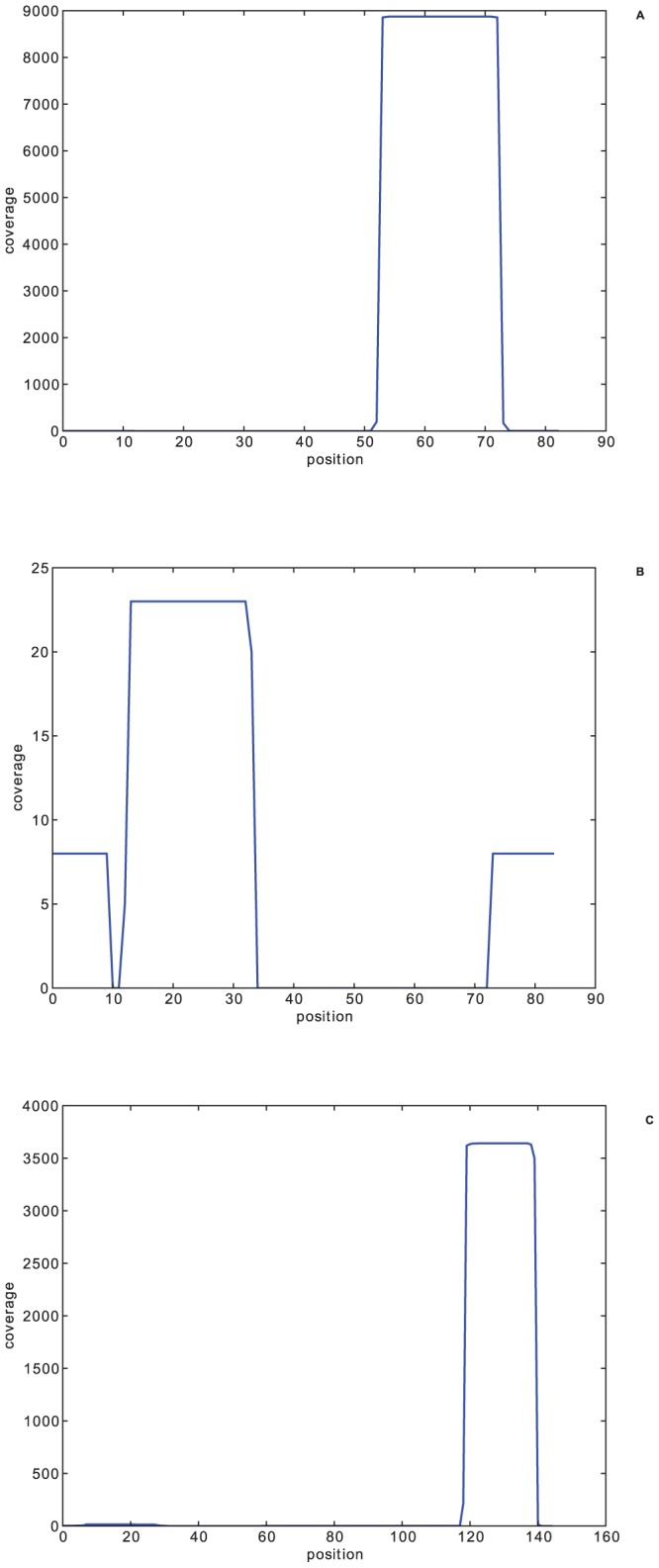
Reads sequenced from pre-miRNAs cannot be assembled into contigs. Reads sequenced from pre-miRNAs cannot be assembled into contigs. Three different miRNAs show highly different expression levels in the same RNA-seq data. A. mir-156 B. mir-160 C. mir-166.

We propose a method that combines homology search and family-specific *de novo* assembly to identify reads sequenced from ncRNAs. In particular, the homology search is applied to both the short reads and contigs produced by assembly programs. This method is designed based on two key observations. First, reads sequenced from ncRNAs tend to share higher sequence and structural similarity with the their native families than reads sequenced from other families. Thus, higher alignment scores by ncRNA homology search tools are expected. In particular, homology search is vital for identifying ncRNAs that go through cleavage and degradation. Reads sequenced from miRNAs are hard to assemble because only reads corresponding to mature miRNAs can be largely captured into RNA-seq data. None or a few can be mapped to other regions of the pre-miRNA due to fast degradation. Figure 1 shows the mapping results of reads sequenced from pre-miRNAs obtained from Arabidopsis. No contig or very short contigs can be produced based on the typical read mapping pattern. In addition, this read mapping pattern does not change with increase of expression levels, as shown in the three miRNAs in Figure 1. For these types of ncRNAs, applying homology search on short reads directly is indispensable.

While homology search is important, applying it to short and fragmentary reads may introduce high false positive rate when detecting remote ncRNA homologs (data will be shown in Methods Section). Thus RNA-CODE employs the second observation that true ncRNA reads sequenced from the same gene can be assembled into contigs with significantly high alignment scores against their native families. On the contrary, reads aligned by chance are not likely to be assembled because they tend to share poor overlaps. Both properties are important in boosting sensitivity and accuracy of short reads classification.

RNA-CODE consists of three key stages. First, RNA-CODE coarsely classifies reads into different ncRNA families using both secondary structure and sequence similarity. Then, a family-specific sequence assembly is used to assemble aligned reads into contigs. Because the numbers of reads that are coarsely classified in the first step indicate the expression levels or abundance of ncRNA genes in this family, this step chooses *de novo* assembly parameters (such as kmer size or overlap threshold) accordingly. The produced contigs are generally longer than input reads and thus can be classified into ncRNA families with better sensitivity and accuracy in the last step. For miRNAs which cannot be assembled into contigs, we use their biogenesis-based property and homology search results for classification.

**Figure 2:**
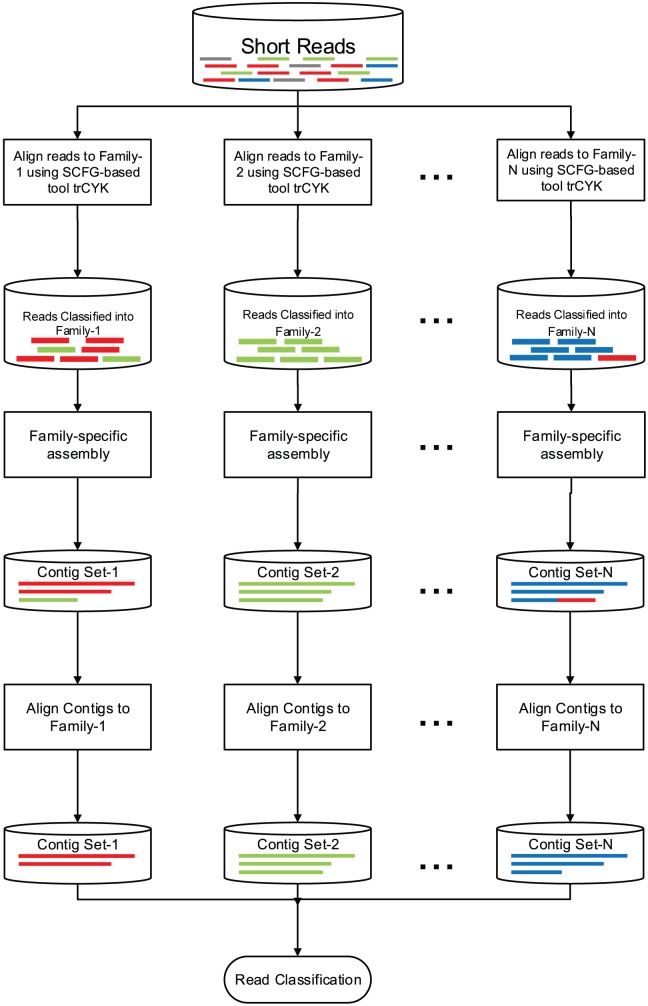
The pipeline of RNA-CODE. The pipeline of RNA-CODE. For miRNAs, the output of the first stage (SCFG-based filtration) and the whole pipeline will be used together for reads classification.

The three-stage workflow with chosen tool for each stage is illustrated in Figure 2. Here, we highlight the rationale behind the design of the three stages. The first stage aims to classify a large number of input reads into different ncRNA families with high sensitivity. It employs existing homology search tools. For short and fragmentary reads, this stage can incur high FP rate. Thus, downstream analysis is needed to remove those falsely classified reads. In the second stage, *de novo* sequence assembly tools are employed to assembly classified reads into contigs. The family-specific sequence assembly is expected to produce contigs corresponding to complete or partial ncRNA genes. However, because of the extremely uneven or low transcriptional levels of many types of ncRNAs or low abundance, a small overlap cutoff or kmer is needed to ensure appropriate connectivity for some families. As a result, some contigs are chimeric or simply consist of randomly aligned reads. The third stage is used to remove the false positives. All contigs are aligned to ncRNA families. Only ones with scores or lengths above given cutoffs are kept. For miRNAs that cannot form contigs, we use stringent homology search scores and known biogenesis-related properties as classification criteria. Every stage will be described in great detail below.

### Stage 1: SCFG-based filtration

To maximize classification sensitivity, short reads are aligned to an SCFG model built from an RNA family of interest. An SCFG describes not only primary sequence of an RNA family but also its secondary structure formed by base pair interaction. The state-of-the-art implementation of SCFG model is Covariance Model (CM). The software suite Infernal [11] builds a CM on a family of RNA sequences, and searches for homologs using inside-outside algorithms. A CM in Infernal is implemented as a tree-like structure in which each node models a single base or a base pair. Infernal is able to optimally align a sequence to this tree with the highest possible score. Short reads, however, pose challenges to the search algorithms because they are fragmentary sequences in which nucleotides expected to form base pairs could be missing. Due to missing bases, base pairs that could have been aligned to a base-pair node in a parse tree are not alignable any more. As a result, reads sequenced from this family may not be well aligned to the underlying CM. Truncated-CYK (trCYK) [14] is a specialized tool designed for fragmentary sequence search. It performs local RNA alignment against a CM of interest, recovering base pairs that are possibly missing and would otherwise be base paired. For every alignment, a score is provided by trCYK indicating the goodness of alignment. Homologous reads tend to yield higher scores and longer alignments than random reads.

**Figure 3:**
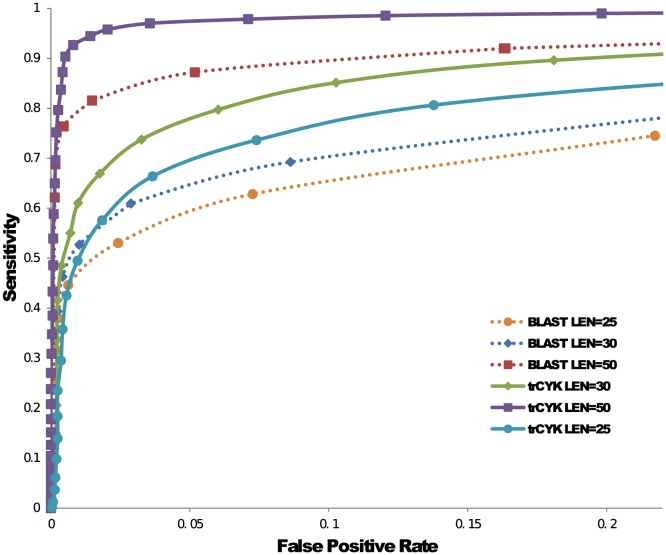
ROC curves of short reads classification using trCYK and BLAST. ROC curves of short reads classification using trCYK and BLAST. Sensitivity measures the ratio of correctly found true tRNAs to the total number of true tRNA reads. False positive rate measures the ratio of falsely found tRNA reads to the total number of false tRNA reads.

Here we report the performance comparison of two homology search tools that can be applied to short and fragmentary reads. One is the mostly commonly used homology search tool BLAST [19], which relies on sequence similarity only. The second tool is trCYK [14]. The goal is to compare the performance of trCYK with BLAST in classifying ncRNA reads of different lengths. Thus, for read length 25, 30 and 50, we sampled 5000 true reads from tRNA sequences obtained from Rfam. Another 5000 random reads generated from other RNA families were mixed with true tRNA reads. Seed sequences from Rfam were excluded from the test data. Covariance Model used in trCYK and formatted database used in BLAST were both built from seed sequences of tRNA. We then searched for tRNA reads in the mixed reads using trCYK and BLAST. The performance of both tools is visualized in the ROC curves in Figure 3. The figure demonstrates that trCYK has better performance than BLAST. However, both tools have high FP rates, showing the need for further screening.

Like all alignment programs, a score cutoff is needed to distinguish homologous sequences from others. We set two cutoffs *s* and *l*, on alignment score and alignment length, respectively. *s* and *l* determine the strength of filtration. A low cutoff will lead to an overwhelming number of negative reads which could significantly slow down the next two stages. A high cutoff, however, will exclude remote homologous reads with poor conservation from further analysis. As trCYK does not provide such thresholds, we considered two strategies to determine the cutoffs. First, the expected alignment score for a homologous sequence of length 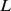 can be used as the cutoff. In order to ensure high sensitivity, the actual cutoff can be smaller than the expected score. Second, Monte-Carlo method can be used to evaluate the sensitivity and FP rate of a score cutoff using a large number of sequences that are generated from both ncRNAs and non-ncRNAs. In this work, we used the second strategy. Figure 3 shows that some very short homologous reads have poor alignment scores. As the first stage defines the upper-bound of the classification sensitivity, we chose a loose cutoff *s* = 1 to guarantee that most positive reads can pass the filtration stage. We found that this threshold also applies to reads sequenced from other types of ncRNAs. With the increase of read length, this score threshold needs to be improved as well. As the first stage is designed to achieve high sensitivity, the default cutoff is set to 1. To further increase the sensitivity of filtration, we also accept reads with alignment score *s* greater than -1 and with alignment length 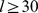 bases.

### Stage 2: family-specific *de novo* assembly

For reads that are coarsely classified to a family by the first stage, they will be input to *de novo* assembly tools. Compared to conducting *de novo* assembly on all the reads, the input sizes to assembly tools are significantly reduced. Thus, even memory intensive assembly tools can be applied.

Multiple *de novo* assembly tools exist. Depending on the data properties, such as read length and sequencing error rates, sensible choices can be made. In this work, the *de novo* assembly programs are applied to RNA-seq data of non-model organisms or metagenomic data. Thus, specific properties of these two data should be considered when choosing assembly tools. Unlike genome assembly, highly diverse sequencing coverage is expected in both data sets. In RNA-seq data, heterogeneous expression levels of ncRNAs contribute to highly diverse sequencing coverage. In metagenomic data, different abundance of ncRNA genes lead to different sequencing coverage. Choosing one set of parameters (such as overlap threshold in overlap graph or kmer size in de Bruijn graph) for the whole data set is not likely to produce optimal results for downstream ncRNA analysis. Thus, the first requirement for the chosen assembly program is that users can adjust the parameters according to the output of the filtration stage. Specifically, although the first stage only coarsely classifies reads into gene families, it can be used to estimate the expression levels or abundance of genes in a family. For families with large number of reads classified, RNA-CODE assumes high sequencing coverage and thus uses stringent assembly parameters. On the other hand, for families with fewer number of classified reads, small overlap cutoffs (in an overlap graph-based assembly tools) or small kmers (in de Bruijn graph) should be used to ensure connectivity for lowly transcribed regions or low abundance genes. Second, many assembly programs removed kmers with low coverage as they may contain sequencing errors. In order to assembly ncRNAs of low expression or abundance, we use an assembly tool that can keep reads/kmers with low coverage.

In this work, for very short reads (read length 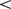
50 bp), we applied and compared several assembly programs [20], [21] and chose SSAKE [22] because it delivers better assembly performance on our experimental data. SSAKE is a specialized *de novo* assembly tool for unpaired short reads assembly. It is a graph-based greedy assembler that efficiently assembles millions of short reads following near-linear time complexity. During the assembly process, the 3′ end of a contig is extended if its suffix overlaps with the prefix of another read. SSAKE generates contigs progressively by searching all prefixes stored in a hash table for the longest possible prefix-suffix match whose overlap is above a threshold. We modified the codes of SSAKE to make it accept any overlap cutoff.

To follow standard notation for assembly algorithms, we use k to represent either the kmer size in De Bruijn graph or the overlap threshold in an overlap graph for assembly. The length of overlap threshold k is an important parameter in SSAKE. A higher k usually results in fewer but more accurate contigs. A lower k leads to higher contiguity. But incorrect extension may happen because the probability of a random prefix-suffix match is high. In *de novo* assembly, there does not exist optimal overlap threshold. Although the expression or abundance is not known a prior, we estimate it using the number of classified reads in the first stage and choose k accordingly. In addition, it is observed that using a single-k will lead to suboptimal performance of *de novo* assemblers. In the second stage, a range of k's are chosen and used on short reads assembly. For each k, all reads are used in assembly. The contigs from different assemblies are subsequently pooled together for further analysis.

### Stage 3: contig selection

**Figure 4:**
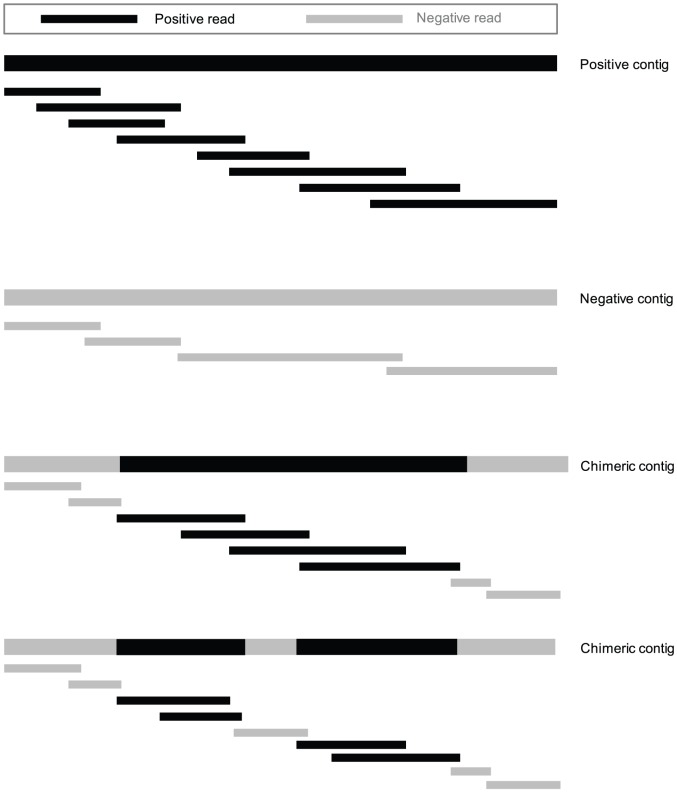
Three types of contigs.

Some randomly aligned reads in stage 1 can be removed by stage 2 because they are not part of any contig. However, due to the loose overlap cutoff or small kmers, some reads can still be assembled into contigs and thus produced by stage 2. There are three types of contigs with reference to an ncRNA gene family: 1) positive contigs that are assembled by reads originated from this family, 2) negative contigs that are assembled from false reads that are not part of the underlying gene family, and 3) chimeric contigs that are formed by both true and false reads. Negative and chimeric contigs can be formed due to small overlaps we allowed in the multiple-k assembly. The probability that a contig is extended with a negative read due to a random prefix-suffix match is high for a small overlap cutoff. Sketches of the three types of contigs are presented in Figure 4.

To distinguish positive contigs from negative ones, we align contigs to the underlying CM. We chose to use trCYK for the following reasons. 1) Both sequence and structural information of a contig should be utilized. 2) Many contigs may not be complete RNA genes especially when the gene transcription level is low. Thus we need to consider missing bases while aligning contigs to the underlying CM.

After trCYK is applied to all contigs from stage 2, if there exist contigs with alignment scores greater than a pre-determined cutoff, the gene of interest is considered to be transcribed. As trCYK is a local alignment tool, it is common that only part of the contig is aligned to the underlying CM. Thus only reads that assemble the aligned part are classified into this RNA family. This feature could be very effective when multiple correct segments exist in a chimeric contig, although we did not observe such cases in our experiment. Bad segments interleaved by correct ones could potentially be removed.

### MIRNA families

Normally, all classified reads need to pass through the entire pipeline. But for miRNA families, as no contigs might be formed, we used two criteria for read classification. First, the alignment score and length of the trCYK alignments in the first stage must pass the pre-determined threshold. Second, for all reads that align to the miRNA* region, we examined whether they can form a stem with reads aligned to the mature miRNA region. If not, the reads aligning to miRNA* region will be removed. As a result, many reads that cannot form any contig can be still classified into miRNAs based only on the homology search results in the first stage.

## Results and Discussion

RNA-CODE can be applied to ncRNA classification in both metagenomic data and RNA-seq data of non-model species. To demonstrate the utility of RNA-CODE, we conducted two experiments. In the first experiment, we tested RNA-CODE on identifying reads sequenced from 16s rRNAs in a small-scale metagenomic data set. It is widely known that 16s rRNA is an important genetic marker for taxonomic identification in metagenomes. Identifying 16s rRNA reads can be used as the first step to assemble full-length 16s rRNAs [4], [6], [7]. This experiment aims to detect reads that are sequenced from 16s rRNAs. In the second experiment, we tested RNA-CODE on annotating reads sequenced from different ncRNA genes including house-keeping RNAs, miRNAs etc. in RNA-seq data of the model organism *Arabidopsis Thaliana*.

For the first experiment, the performance of RNA-CODE was benchmarked with Metaxa [4], which is designed for classifying short reads into different rRNA families. For the second experiment, the performance of RNA-CODE was benchmarked with standard annotation pipeline for NGS data, which is *de novo* assembly tools plus existing ncRNA annotation tools.

To evaluate the performance of all tools, we compared the true membership and the predicted membership of reads. Two metrics are used in evaluation: read-level sensitivity and positive predictive value (PPV), which indicates accuracy. For an RNA family of interest, let 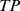 be the set of true positive reads originated from this ncRNA family. Let 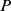 be the set of reads predicted to be positive. Sensitivity is defined as
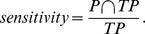



PPV is defined as re appropriate
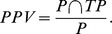



A good ncRNA identification tool should have both high sensitivity and high PPV.

### Detecting reads of 16s ribosomal RNAs

RNA-CODE can detect and discriminate among multiple ncRNA types. This experiment tests RNA-CODE in recognition of one type of ncRNA, 16s rRNA in a metagenomic data set. Various tools exist for 16s rRNA search [4], [11], [23], [24], of which, only Metaxa is designed for short reads. Thus, we benchmark RNA-CODE with Metaxa in this experiment.

#### Data

In order to accurately evaluate the performance of RNA-CODE, we need to know the ground-truth membership of reads in metagenomic data. Thus, we constructed a small-scale real metagenomic data set, for which we knew which reads were sequenced from 16s rRNAs. We obtained human gut microbial metagenomics data from European Nucleotide Archive (http://www.ebi.ac.uk/ena/data/view/ERA000116). The data were sequenced from fecal specimens of obese individual human adults using Illumina Genome Analyser [25]. We selected two pair-ended metagenomics data sets of different read lengths. There were 9,633,603 reads of length 44 in the one dataset and 14,822,431 reads of length 75 in the other. Without knowing the genomes of the species in this sample and their 16s rRNA annotations, we cannot obtain the true membership of all the reads. Thus, we need to construct a small metagenomic data set using reads that can be reliably labeled as 16s rRNAs in fully sequenced genomes. To do this, we first chose species that have whole genomes and 16s rRNA annotations using the species catalog in [26]. Then, all reads that can be mapped to these genomes were included in the small metagenomic data set. In total, 11 strains with complete genomes available were selected from 67 strains designed by Turnbaugh[26]. To determine whether a read was originated from 16s ribosomal genes, we mapped each read back to the genomes of the 11 selected bacteria strains. The read mapping positions and the annotation of 16s rRNA genes are combined to determine whether a read is sequenced from 16s rRNAs. If a mapped read share at least 80% of bases with an underlying 16s genes, we defined this read as positive. If a read has no overlap with any 16s gene, we defined it as negative. The reads having less than 80% of their bases overlapped with a 16s gene are considered to be ambiguous and were discarded. Additionally, the reads that cannot be mapped to any of the 11 genomes were also discarded, because we did not have enough information of their true membership. The small-scale metagenomic data consists of both positive and negative reads. For the data set of read length 44, there are 606 positive reads and 71993 negative reads, respectively. For the data set of read length 75, there are 379 positive reads and 61086 negative reads, respectively.

#### Experimental results

We applied RNA-CODE to the small-scale metagenomic data sets constructed above for 16s rRNA classification. We then compared RNA-CODE with Metaxa, a specialized tool for detecting reads originated from SSU rRNAs such as 12s, 16s and 18s. Same as RNA-CODE, input to Metaxa is also a set of short reads in FASTA format. Output of Metaxa contains short reads presumably originated from SSU rRNAs. Short reads are categorized by the species or organelles that they are originated from. The categories include bacteria, archaea, eukaryota, mitochondria, and chloroplast. Because specific categories of reads are not concerned in this experiment, we considered a read to be a true positive if it can be categorized in any of the 5 categories.

The above experiments were conducted on reads of 44 bases and 75 bases using default parameters. As displayed in Table 1, both tools achieved high specificity for both read lengths. The sensitivity of RNA-CODE out-performed Metaxa for both read lengths. Specifically, RNA-CODE performed well on shorter reads. As Metaxa does read classification using hidden Markov models (HMMs), short reads tend to produce marginal scores and thus are hard to distinguish from non-rRNA reads. RNA-CODE considers both sequence and secondary structure similarity and is more sensitive for short read classification. For the same reason, as Metaxa relies on HMMs, it is not expected to perform well on other types of ncRNAs that lack strong sequence similarity.

**Table 1:** Performance comparison of RNA-CODE vs Metaxa. Both tools were applied using the default parameters.

	RNA-CODE		Metaxa	
read length	sen	PPV	sen	PPV
44	0.999	1.000	0.786	1.000
75	1.000	1.000	0.986	1.000

### NCRNA classification in RNA-seq data

To demonstrate the utility of RNA-CODE on detecting reads sequenced from various ncRNA genes, we conducted the second experiment on real RNA-seq data. RNA-CODE classifies reads into different ncRNA families. For house-keeping ncRNAs such as tRNAs and rRNAs, which contain multiple gene members, the number of classified reads show the overall expression levels of this type of ncRNA. For single-member ncRNA families such as many miRNA families in some species, the number of classified reads quantifies the expression level of this gene. We chose to use RNA-seq of the model species *Arabidopsis Thaliana*, which has high-quality genome assembly and gene annotation available in TAIR 10 (http://www.arabidopsis.org), enabling us to determine the true membership of reads with high confidence.

#### Data

An RNA-seq dataset obtained from NCBI SRA (accession number GSM706704) was used in this experiment. This dataset was sampled from transcriptome of inflorescence tissues of *Arabidopsis Thaliana*. The sample was sequenced using Illumina platform and contains 2,327,100 short reads. After removing adaptor sequences and quality trimming, the average length of reads is 23.5, which poses a great challenge for both homology search tools and *de novo* assembly tools. Using ncRNA annotation from TAIR 10, there are hundreds of ncRNAs annotated in Arabidopsis. In this work, we present the results on classifying reads into ncRNAs annotated on chromosome 2 of Arabidopsis.

According to TAIR10 annotation and read mapping results, there are 15 transcribed ncRNAs on chromosome 2 in this RNA-seq data. Out of the 15, there is one miRNA mir-825a that does not have corresponding family in Rfam. In addition, miRBase shows that there are only two sequences annotated as mir-825, which are not enough to train a model. Thus, we excluded this miRNA from our test. After removing this family, we had in total 14 transcribed ncRNAs on chromosome 2 in this data set. These families were used to evaluate the sensitivity of ncRNA classification. The number of mapped reads for the 14 ncRNA families can be found in Table 2. In order to evaluate both the sensitivity and accuracy of RNA-CODE, we randomly chose 32 non-transcribed but annotated ncRNA families as negative test data. The non-transcribed families have zero mapped read and are used to evaluate the accuracy of RNA-CODE. Ideally, an accurate ncRNA detection tool should not classify any read in this RNA-seq data into these ncRNAs.

**Table 2:** Number of reads that are mapped to chromosome 2 of Arabidopsis.

ID in Rfam	gene name	num of mapped reads	num mapped reads(unique)
RF00002	5.8S ribosomal RNA	27984	417
RF00005	tRNA	35602	1339
RF00055	Small nucleolar RNA SNORD96	29	12
RF00073	mir-156 microRNA precursor	8875	13
RF00075	Mir-166 microRNA precursor	3657	18
RF00247	Mir-160 microRNA precursor	31	4
RF00268	Small nucleolar RNA snoZ7/snoR77	7	5
RF00300	Small nucleolar RNA Z221/R21b	79	13
RF00452	mir-172 microRNA precursor	101581	15
RF00647	microRNA MIR164	2746	5
RF00689	microRNA MIR390	933	11
RF00690	microRNA MIR408	322	12
RF00893	microRNA MIR854	1367	313
RF01280	Small nucleolar RNA snoR14	9	8

For each of the 46 ncRNA families (14 positive +32 negative), we used the corresponding SCFG-based models in Rfam 10.1 as input to RNA-CODE. Read mapping results and TAIR10 annotation are used to determine the membership of reads. Only if a read has more than 80% of its bases overlapping with an annotated gene, we consider the read to be a member of this gene. Reads with no overlapping bases are unlikely to be valid transcripts of the gene of interest. Such reads are considered to be negative.

#### Experimental results

We evaluate the performance of RNA-CODE from four aspects. First, as the filtration stage of using trCYK is important to the performance of RNA-CODE, we analyze the performance of trCYK in this experiment. Second, we compare the performance of RNA-CODE with *de novo* assembly tools. Third, we demonstrate the utility of using multiple overlap thresholds in RNA-CODE. Finally, we present case studies for miRNA genes, which produce reads that share no overlaps.

#### Performance of filtration

In this experiment, we report the performance of the filtration stage for different types of ncRNAs. The first stage of RNA-CODE uses trCYK to coarsely classify reads into different families. Only reads that pass this filtration stage will be further analyzed. Thus, the filtration stage determined the upper bound of the sensitivity of RNA-CODE. A low score cutoff is used to keep high sensitivity. The price paid, however, is low specificity, as displayed in Table 3. trCYK did not show good discriminative power on some genes because the transcripts sequenced from these genes are not well conserved and cannot form statistically significant alignments with the underlying CM. For example, a majority of transcripts originated from *Small nucleolar RNA Z221/R21b* had alignment scores lower than the defined threshold due to poor conservation. Thus most reads cannot pass the filtration.

**Table 3:** Filtration statistics.

gene name	sensitivity	PPV
5.8S ribosomal RNA	0.891	0.415
tRNA	0.882	0.5
Small nucleolar RNA SNORD96	1	0.018
mir-156 microRNA precursor	1	0.039
Mir-166 microRNA precursor	1	0.007
Mir-160 microRNA precursor	1	0.012
Small nucleolar RNA snoZ7/snoR77	1	0.003
Small nucleolar RNA Z221/R21b	0.077	0.001
mir-172 microRNA precursor	1	0.015
microRNA MIR164	1	0.002
microRNA MIR390	1	0.011
microRNA MIR408	1	0.018
microRNA MIR854	0.721	0.447
Small nucleolar RNA snoR14	1	0.007

#### Performance comparison with SSAKE

Reads that are classified into each family are used as input to *de novo* assembly programs. For N input families, N *de novo* assembly programs can be run in parallel. A number of *de novo* assembly tools are available. However, due to short read length and low coverage for many types of ncRNAs, some popular tools such as Velvet [26] only produces a few contigs. We empirically compared several *de novo* assembly tools and chose SSAKE 3.8 for two reasons. First, it produced more contigs than others. Second, the source codes of SSAKE is relatively easy to modify to address specific needs for this data. SSAKE was designed for short reads assembly and the minimum length of an input read is 22 bases. In this data, there exist reads of less than 22 bases. So we customized SSAKE to make it accept reads as short as 19 bases. SSAKE requires a minimum overlap of 16 bases when extending contigs. When gene expression level is low, the overlap between positive reads is often lower than 16 bases. To assemble reads of lowly transcribed genes, we customized SSAKE to extend a contig when its suffix has more than 5 overlapping bases with a prefix of a read. We set *Minimum Number of Reads Needed to Call a Base During an Extension* to be 1, as opposed to 2, the default value. The rationale of using 1 is that in the transcripts of poorly expressed genes, base coverage is low. It is rare to find duplicates of a read when extending with this read.

The chosen assembly tool is run for each family separately. Thus, family-specific assembly parameters can be chosen. In particular, the overlap threshold of SSAKE can be adjusted according to the number of classified reads by trCYK. Although the number of classified reads is not an accurate indication of depth of read coverage, it is preferred to choose a small overlap threshold if the number of classified reads is small. In addition, it has been shown that using multiple kmers can improve RNA-seq assembly [21]. As described in our method, RNA-CODE also uses multiple overlap thresholds. Thus, for SSAKE, we allow overlap from 6 to 16 if the number of classified reads by trCYK is less than 10,000. Otherwise, we use 10 to 20. Users can adjust these parameters according to any known knowledge.

The performance of RNA-CODE on the 14 transcribed families is listed in Table 4. For the 32 control families, which were not considered to be transcribed, RNA-CODE did not find any reads, yielding 0% false positive rate. This indicates that RNA-CODE can successfully distinguish transcribed families from un-transcribed ones. Table 4 also includes the performance comparison with SSAKE. More specifically, all reads are used as input to SSAKE. Then, all contigs produced by SSAKE are searched against input ncRNA families using trCYK. The comparison shows that RNA-CODE yielded better performance on all genes except for *5.8S ribosomal RNA*. Without the first stage, a large number of negative reads may be assembled together with positive reads and form chimeric contigs. In chimeric contigs, positive reads could be interleaved by negative reads, making the alignment score between itself and the underlying CM low.

**Table 4:** Performance of RNA-CODE (multiple-k), SSAKE, and RNA-CODE (single-k) on transcribed ncRNA families.

gene name	RNA-CODE		SSAKE		single-k	
	sen	PPV	sen	PPV	sen	PPV
5.8S ribosomal RNA	0.778	0.95	0.99	0.884	0.999	0.978
tRNA	0.437	0.984	0.274	0.996	0.044	1
**Small nucleolar RNA SNORD96**	1	1	0.857	1	0.903	1
**mir-156 microRNA precursor**	0.929	1	0.786	1	0	N/A
Mir-166 microRNA precursor	1	0.947	0.944	1	0.996	1
**Mir-160 microRNA precursor**	0.75	0.214	0	N/A	0	N/A
**Small nucleolar RNA snoZ7/snoR77**	1	1	0	N/A	0	N/A
Small nucleolar RNA Z221/R21b	0	N/A	0	N/A	0	N/A
**mir-172 microRNA precursor**	1	1	0.733	1	1	1
**microRNA MIR164**	1	1	1	1	1	1
microRNA MIR390	0.909	1	0.909	1	0.996	1
microRNA MIR408	1	0.75	1	1	1	1
microRNA MIR854	0.599	0.978	0.468	0.698	0.415	1
Small nucleolar RNA snoR14	0.25	1	0	N/A	0	N/A

The reason why RNA-CODE was out-performed on *5.8S ribosomal RNA* is that many short reads originated from *5.8S ribosomal RNA* did not pass the filtration due to poor conservation. As a result, the overall sensitivity is lower than *de novo* assembly tools.

#### Using multiple overlap thresholds improves performance of RNA-CODE

Multiple-k approach is another important component in the pipeline. To demonstrate the effectiveness of this approach, we compared RNA-CODE using multiple-k and single-k approach. Sensitivity of RNA-CODE using multiple-k is generally better than using single-k, except for *5.8S ribosomal RNA* and *MIR-390*. We used the default k value (i.e. 16) in single-k approach. Many contigs assembled from short reads originated from lowly expressed genes were not recovered in the single-k approach; since overlap between two positive reads may be less than 16 bases. However, using multiple overlap thresholds may also introduce chimeric contigs, which explains the worse performance of RNA-CODE using multiple k than single k on *5.8S ribosomal RNA* and *MIR-390*.

#### Performance of microRNA families

For eight transcribed miRNA genes, RNA-CODE performs better in six of them and has the same sensitivity and PPV for the other two. This is expected because miRNA reads usually cannot be assembled. In this data set, because of the extreme short reads, some of them are assembled into the mature miRNA and thus are able to be output by SSAKE. For example, reads of length between 19 and 22 are assembled into the mature miRNA of length 25 for mir-156. For most RNA-seq data that have longer reads after quality trimming, *de novo* assembly tools will not be able to assemble them into contigs. Thus, using both homology search and *de novo* assembly is important to generate a comprehensive catalog of ncRNAs.

## Conclusion

We presented an ncRNA classification tool that can determine the membership of reads that are sequenced from ncRNA genes. By combining homology-based ncRNA search method and family-specific *de novo* assembly, we can classify reads into different types of ncRNAs, including those that cannot be assembled because of cleavage and degradation. This tool can be applied to NGS data that do not have quality reference genomes, such as metagenomic data and RNA-seq data of non-model organisms.

RNA-CODE relies on trCYK as the ncRNA homology search tool for very short reads. When reads are longer, more efficient ncRNA homology search tool such as Infernal [11] can replace trCYK. For very short reads, trCYK is still the best choice in order to yield high sensitivity.
